# Pressured HIV testing “in the name of love”: a mixed methods analysis of pressured HIV testing among men who have sex with men in China

**DOI:** 10.1002/jia2.25098

**Published:** 2018-03-25

**Authors:** Jason J Ong, Dan Wu, Wenting Huang, Hongyun Fu, Nicola Desmond, Wei Ma, Dianmin Kang, Meizhen Liao, Gifty Marley, Chongyi Wei, Weiming Tang, Chuncheng Liu, Ye Zhang, Stephen W Pan, Bin Yang, Ligang Yang, Shujie Huang, Joseph D Tucker

**Affiliations:** ^1^ Department of Global Health and Development London School of Hygiene and Tropical Medicine London United Kingdom; ^2^ Social Entrepreneurship to Spur Health (SESH) Global Guangzhou China; ^3^ Central Clinical School Monash University Melbourne VIC Australia; ^4^ Eastern Virginia Medical School Norfolk VA USA; ^5^ Department of International Public Health Liverpool School of Tropical Medicine Liverpool United Kingdom; ^6^ School of Public Health Shandong University Jinan China; ^7^ Shandong Centre for Disease Control and Prevention Jinan China; ^8^ Rutgers School of Public Health New Brunswick NJ USA; ^9^ University of North Carolina at Chapel Hill North Carolina NC USA; ^10^ Southern Medical University Dermatology Hospital Guangzhou China; ^11^ Faculty of Infectious and Tropical Diseases London School of Hygiene and Tropical Medicine London UK

**Keywords:** HIV, coercion, testing, men who have sex with men, China, mixed methods

## Abstract

**Introduction:**

HIV testing has rapidly expanded into diverse, decentralized settings. While increasing accessibility to HIV testing is beneficial, it may lead to unintended consequences such as being pressured to test. We examined the frequency, correlates and contexts of pressured HIV testing among Chinese men who have sex with men (MSM) using mixed methods.

**Methods:**

We conducted an online survey of MSM (N* *=* *1044) in May 2017. Pressured HIV testing was defined as being forced to test for HIV. We conducted logistic regression analysis to determine the associations between pressured HIV testing and socio‐demographic and sexual behavioural factors. Follow‐up interviews (n = 17) were conducted with men who reported pressured testing and we analysed qualitative data using a thematic analysis approach.

**Results:**

Ninety‐six men (9.2%) reported experiencing pressure to test for HIV. Regular male sex partners were the most common source of pressure (61%, 59/96), and the most common form of pressure was a threat to end a relationship with the one who was being pressured (39%, 37/96). We found a higher risk of pressured testing in men who had only used HIV self‐testing compared to men who had never self‐tested (AOR 2.39 (95%CI: 1.38 to 4.14)). However, this relationship was only significant among men with low education (AOR 5.88 (95% CI: 1.92 to 17.99)) and not among men with high education (AOR 1.62 (95% CI: 0.85 to 3.10)). After pressured testing, about half of men subsequently tested for HIV (55%, 53/96) without pressure – none reported being diagnosed with HIV. Consistent with this finding, qualitative data suggest that perceptions of pressure existed on a continuum and depended on the relationship status of the one who pressured them. Although being pressured to test was accompanied by negative feelings, men who were pressured into testing often changed their attitude towards HIV testing, testing behaviours, sexual behaviours and relationship with the one who pressured them to test.

**Conclusion:**

Pressured HIV testing was reported among Chinese MSM, especially from men with low education levels and men who received HIV self‐testing. However, in some circumstances, pressure to test helped MSM in several ways, challenging our understanding of the role of agency in the setting of HIV testing.

## 1 Introduction

Men who have sex with men (MSM) in China are estimated to account for a third of new HIV infections each year 1. Increasing the coverage and frequency of HIV testing among MSM is an essential strategy to curtail the spread of HIV and is actively promoted by all levels of the Chinese government. Free facility‐based HIV testing services are available for MSM at community‐based voluntary counselling and testing (VCT) sites, centres of disease control (CDCs) and public hospitals. In addition, HIV self‐testing, which increases testing agency with regard to when, where and around whom testing occurs 2, has been scaled up nationwide and is gaining traction among MSM 3. It is estimated that between 61% to 87% of Chinese MSM living with HIV are undiagnosed 4, 5. With the help of educational campaigns and other social marketing approaches to improve awareness in MSM, lifetime HIV testing uptake has significantly increased from 24% to 47% among MSM between 2006 and 2011, and testing in the preceding 12 months had also increased from 21% to 38% respectively 6. While this increase in testing is undoubtably a public health benefit, there may be unintended consequences of widespread testing.

One potential outcome of a massive scale‐up in HIV testing is unwanted pressure to test for HIV. Pressured testing is defined as being forced to take an HIV test against one's will through verbal, physical or psychological threat 7. For example, pressure may occur through physical force, or it may involve threats if one refuses to test (e.g. terminating employment, ending a relationship, withholding sex) 8. The World Health Organization (WHO) and The Joint United Nations Programme on HIV/AIDS (UNAIDS) HIV self‐testing guidelines opposes coerced HIV test in any setting including from sexual partners and family members 2. All recipients of an HIV test must undergo informed consent; more importantly, everyone is entitled to the right to refuse an HIV test. However, negotiated safety within the context of sexual relationships is also critical, and individuals should be empowered to openly and effectively communicate strategies to reduce HIV acquisition, without the need to resort to inappropriate strategies to pressure someone else to test for HIV 9.

Despite these guidelines, HIV test pressure has been reported. For example, qualitative interviews conducted in Malawi found pressured testing between heterosexual couples 10. Pressure occurred in a complex social context and pressured testing was considered by some Malawians as an acceptable and beneficial way to increase agency (i.e. a sense of control) in their lives 10. In China, there have been reports of pressured testing in sex workers and drug users in detention settings 11, 12, 13. There are reports of intimate partner violence and coercion to participate in studies within MSM couples 14. However, little is known about the frequency of HIV test pressure amongst MSM. Specifically, it is unclear what forms of pressure may be occurring, how men perceive pressure, which factors contribute to pressure and what consequences may result from HIV test pressure among MSM. To address this knowledge gap, we used data from a quantitative survey as well as in‐depth interviews to examine the frequency, correlates and contexts of pressured HIV testing among MSM in China.

## 2 Methods

### 2.1 Study design and setting

An online survey of 1044 MSM was conducted in May 2017, as part of a randomized control trial among MSM living in China. Men were recruited from eight cities ‐ four from Guangdong Province (Guangzhou, Jiangmen, Zhuhai and Shenzhen) and four from Shandong Province (Yantai, Jinan, Qingdao, and Jining) in China. These cities were chosen based on having a CDC MSM sentinel surveillance site and local capacity for implementing HIV interventions. Men recruited into the trial were born biologically male, were aged 16 years and older, self‐reported as HIV‐negative, and had oral or anal sex with another man at least once during their lifetime.

### 2.2 Measures

Demographic data were collected, including the following: age (continuous), household registration (rural vs. urban), whether they were students (yes/no), marital status (ever married/not married), highest level of education (high school or below/college or above), annual income (<2693, 2693 to 14,361, >14,361 USD) and city of recruitment.

Sexual health and behavioural measures: We collected self‐identified sexual orientation (gay, non‐gay), disclosure of sexual orientation to people other than their sexual partner (yes/no) or health professional (yes/no), main settings for meeting sexual partners (online/offline), anal sex in the last 3 months (yes/no), condomless anal sex in the last 3 months (yes/no), and vaginal/anal sex with a female ever (yes/no).

*Self‐efficacy score*: We calculated a mean self‐efficacy score based on a six‐item scale adapted from Gu et al. 15, which were answered on a four‐point Likert‐scale ranging from strongly disagree to strongly agree. A higher mean score represents higher HIV testing self‐efficacy.

*Community engagement*: We evaluated the responses to six questions, then categorized community engagement as none, minimal, moderate or substantial engagement 16.

*HIV stigma*: We assessed the response to seven questions, then presented the mean score on a scale between 1 to 4, with higher scores indicating greater HIV stigma 17.

#### 2.2.1 Quantitative assessment of HIV test pressure

We asked participants whether they had ever experienced HIV test pressure, defined as being forced against their will to take an HIV test. We further categorized pressure as physical violence (e.g. pushing, slapping, punching, kicks), threats of violence, verbal abuse (e.g. being shouted at), psychological pressure (e.g. being neglected, being discriminated), excessive control of activities (e.g. not being allowed to leave the house), withholding of household resources, and threats to end a relationship. We also collected data on last experienced HIV test pressure (within 3 months, between 3 months to a year ago, more than a year ago), lifetime occurrences of pressure, the setting and who pressured them to test. We asked whether the person who pressured them was present at the time of testing, and whether the participant told anyone else about their experience of pressure. We also asked about the effect of pressure on their likelihood to test for HIV and their HIV testing history since their last experience of test pressure.

#### 2.2.2 Semi‐structured individual interviews: qualitative assessment of HIV test pressure

We invited all men who reported pressured testing in their survey results to participate in a follow‐up telephone interview. We conducted semi‐structured interviews exploring categories of pressured testing, context(s) of the pressured testing, impact of the pressured testing on the participant, and their attitudes towards HIV testing. The inclusion criteria was men who reported at least one of the following forms of pressure: physical violence, threats of violence, verbal abuse, psychological pressure, excessive control of activities, withholding of household resources or threats to end a relationship. A research assistant sent an online invitation message to eligible participants, and a follow‐up call was made to confirm participation. Two researchers experienced in qualitative research conducted the interviews. We obtained verbal consent before the interview, stressing confidentiality and voluntary participation. Phone interviews were conducted between May and August 2017 and each lasted between 45 and 60 minutes. An incentive of 7.50 USD (50 Chinese Yuan) mobile phone top‐up was provided for participants.

### 2.3 Data analysis

#### 2.3.1 Quantitative survey data analysis

Descriptive statistics were reported to summarize the socio‐demographic characteristics of the study population and men who had reported experiencing HIV test pressure. We also examined the category of pressure experienced according to the relationship with the one who pressured them. Bivariable and multivariable logistic regression were conducted to identify factors associated with HIV test pressure experience. We included independent variables with a univariable analysis *p* value of less than 0.25 in the multivariable models^18^. The importance of each variable was assessed using a *p* value of its Wald statistic. Variables that were statistically insignificant (*p* > 0.05) were sequentially eliminated from the model. We tested for significant interactions between HIV testing history and age, income, education level, city of recruitment, household registration, sexual identity, disclosure of sexual identity to others and health professionals, condomless anal sex, sex with female partners, self‐efficacy, community engagement and stigma. We included the interaction term into the final model if *p* < 0.05. The Hosmer‐Lemeshow test was used to assess goodness of fit. Data were analysed using STATA (StataCorp. 2013. *Stata Statistical Software: Release 13*. College Station, TX: StataCorp LP).

Qualitative data were audio recorded and transcribed in Chinese; relevant quotations were translated into English for this manuscript. A research assistant checked the accuracy of the transcripts. We analyzed the data using a thematic approach with the assistance of NVivo 11. An initial codebook was established based on three transcripts and we added new codes when new codes were identified or existing codes warranted modification. Two researchers (DW, WT) coded all transcripts independently based on the codebook and checked for consistency and accuracy. DW, WT and JJO discussed the codes. Afterward, we organized and categorized codes into different themes/subthemes and further explored relevant themes in subsequent interviews. We continued data collection until thematic saturation was reached.

Ethics approval was obtained from the ethics review committees at the Guangdong Provincial Center for Skin Diseases and STI Control, the University of North Carolina at Chapel Hill, and the University of California, San Francisco.

## 3 Results

### 3.1 Experience of HIV test pressure – quantitative results

Of 1044 men who completed the survey, 96 men reported ever experiencing HIV test pressure (9.2%, 95% confidence interval [7.5% to 11.1%]). Table 1 summarizes the demographics and sexual behaviours of MSM who reported HIV test pressure. They had a mean age of 25.8 (standard deviation 6.4). The majority self‐identified as gay (69%), met sexual partners mainly online (79%) and had anal sex with a male partner in the preceding three months (69%).

**Table 1:** Demographics and sexual behaviours of men who have sex with men who reported HIV test pressure in China (*N = *1044), 2017

	Total study population (N = 1044)	Men with pressured HIV testing (N = 96) n (%)	No pressured HIV testing (N = 948) n (%)
Demographics			
Mean age ± SD	25.4 ± 6.5	25.8 ± 6.4	25.3 ± 6.5
Rural household registration	482 (46.2)	42 (43.8)	440 (46.4)
Student	280 (26.8)	24 (25.0)	256 (27.0)
Ever married	91 (8.7)	9 (9.4)	82 (8.7)
Highest level of education – high school or below	364 (34.9)	30 (31.3)	334 (35.2)
Annual income (USD)			
<2693	225 (21.6)	20 (20.8)	205 (21.6)
2693 to 14,361	724 (69.3)	66 (68.8)	658 (69.4)
>14,361	95 (9.1)	10 (10.4)	85 (9.0)
City of Recruitment			
Guangzhou	156 (14.9)	18 (18.8)	138 (14.6)
Shenzhen	160 (15.3)	15 (15.6)	145 (15.3)
Zhuhai	110 (10.5)	7 (7.3)	103 (10.9)
Jiangmen	107 (10.3)	16 (16.7)	91 (9.6)
Jinan	130 (12.5)	12 (12.5)	118 (12.5)
Qingdao	135 (12.9)	10 (10.4)	125 (13.2)
Yantai	120 (11.5)	8 (8.3)	112 (11.8)
Jining	126 (12.1)	10 (10.4)	116 (12.2)
Sexual behaviours			
Gay sexual identity	749 (71.7)	66 (68.8)	683 (72.1)
Disclosure of sexuality/sexual history to others	682 (65.3)	63 (65.6)	619 (65.3)
Disclosure of sexuality/sexual history to health professional	211 (20.2)	18 (18.8)	193 (20.4)
Met sexual partners mainly online	778 (74.5)	76 (79.2)	702 (74.1)
Anal sex with a male in the last 3 months	613 (58.7)	66 (68.8)	547 (57.7)
Condomless sex in the last 3 months	253 (24.2)	30 (31.3)	223 (23.5)
Ever had vaginal or anal sex with female partner	223 (21.4)	30 (31.3)	193 (20.4)
Mean self‐efficacy score ± SD	1.9 ± 0.5	1.9 ± 0.6	1.9 ± 0.5
Community Engagement in sexual health^#^			
None	143 (13.7)	10 (10.4)	133 (14.0)
Minimal	115 (11.0)	4 (4.2)	111 (11.7)
Moderate	509 (48.8)	51 (53.1)	458 (48.3)
Substantial	277 (26.5)	31 (32.3)	246 (26.0)
Mean anticipated HIV stigma Score ± SD	2.0 ± 0.6	2.0 ± 0.7	2.0 ± 0.6
HIV testing history			
Never tested	456 (43.7)	27 (28.1)	429 (45.3)
Ever self‐tested	222 (21.3)	29 (30.2)	193 (20.4)
Ever facility tested	111 (10.6)	7 (7.3)	104 (11.0)
Both self‐ and facility test	255 (24.4)	33 (34.4)	222 (23.4)

Community engagement was based on responses to six questions and then categorized into four groups (see ref. 16).

SD = standard deviation.

Table 2 is a summary of the forms of pressure experienced, according to the relationship with the person who pressured them. The most common person who pressured them were regular male sexual partners (61%, 59/96), casual male sexual partners (29%, 28/96) and friends (24%, 23/96). Threatening to end a relationship was the most common form of pressure for regular and casual sexual partners, parents, and educational institutions. In contrast, psychological pressure was most commonly used by friends, health workers and family members other than parents. Participants reported last experiencing pressured testing within the last 3 months, (28%, 27/96), between 3 months to 1 year ago (39%, 37/96), and more than a year ago (33%, 32/96). The most common settings of pressure were the participant's homes (45%, 43/96), health facilities (19%, 18/96), other people's home (18%, 17/96), the participant's workplaces (15%, 14/96), hotels (13%, 12/96), community based organizations (10%, 10/96), educational institutions (8%, 8/96), entertainment settings (8%, 8/96) and prison/detainment centres (2%, 2/96). Most men reported more than one episode of HIV test pressure (73%, 55/75), with a median of 2 [IQR 1 to 3] episodes of pressure in their lifetime.

**Table 2:** The forms of pressure experienced according to who pressured them, in men who have sex with men in China *(N *=* *96), 2017

	Physically hurt n(%)	Threats of being physically hurt n(%)	Verbal insult n(%)	Psychological pressure n(%)	Being controlled n(%)	Denying access to household resources n(%)	Threatening to end a relationship n(%)
Source of pressure							
Regular male sexual partner (n = 59)	6 (10)	4 (7)	7 (12)	18 (31)	7 (12)	3 (5)	27 (46)
Casual male sexual partner (n = 28)	1 (4)	2 (7)	5 (18)	11 (39)	3 (11)	1 (4)	13 (46)
Friends (n = 23)	1 (4)	1 (4)	8 (35)	10 (43)	5 (22)	2 (9)	6 (26)
Healthcare worker (n = 9)	1 (11)	0 (0)	2 (22)	4 (44)	1 (11)	0 (0)	2 (22)
Other Family (n = 8)	1 (13)	1 (13)	2 (25)	3 (38)	0 (0)	2 (25)	1 (13)
Parents (n = 5)	1 (20)	0 (0)	0 (0)	1 (20)	1 (20)	0 (0)	2 (40)
Person from an educational institution (n = 5)	0 (0)	0 (0)	1 (20)	2 (40)	0 (0)	0 (0)	4 (80)
Person from the government (n = 2)	0 (0)	0 (0)	0 (0)	1 (50)	1 (50)	0 (0)	0 (0)
Boss (n = 1)	0 (0)	0 (0)	0 (0)	1 (100)	0 (0)	0 (0)	0 (0)

### 3.2 Consequences of HIV test pressure – quantitative results

Sixty‐nine out of 96 (72%) men tested for HIV as a result of the pressure: two men tested positive. The majority of men reported that the person who pressured them was present at the time of testing (80%, 55/69). Only a minority told someone else about their experience of pressure (24%, 23/96). After their experience of pressured testing, about half of men tested for HIV again without any pressure (55%, 53/96) and all reported testing negative for HIV. Of those who had not tested again after being coerced, five men reported that their experience of pressure made them less likely to test again.

Table 3 summarizes the results from the logistic regression analysis. In the bivariable analysis, we found that HIV test pressure was more likely in men who had self‐tested only (OR 2.39 (95% CI: 1.38 to 4.14), had both self‐tested and facility‐tested (OR 2.36 (95% CI: 1.38 to 4.03), and in men who had ever had sex with females (OR 1.78 (95% CI: 1.12 to 2.82). Among the interactions examined, effect modification was noted only for education level (Table 4). We found that higher education (i.e. college level or above) buffered the increased odds of HIV test pressure in men who self‐tested.

**Table 3:** Bivariable and multivariable logistic regression models of men who have sex with men who ever experienced HIV test pressure in China (N* *=* *1044)*,* 2017

	Crude odds ratio (95% CI)	*p* value	AOR* (95% CI)	*p* value
City of recruitment				
Guangzhou	1	–	–	
Shenzhen	0.79 (0.38 to 1.64)	0.53	–	
Zhuhai	0.52 (0.21 to 1.29)	0.16	–	
Jiangmen	1.35 (0.65 to 2.78)	0.42	–	
Jinan	0.78 (0.36 to 1.69)	0.53	–	
Qingdao	0.61 (0.27 to 1.38)	0.24	–	
Yantai	0.55 (0.23 to 1.31)	0.18	–	
Jining	0.66 (0.29 to 1.49)	0.32	–	
Condomless sex in the last 3 months	1.48 (0.94 to 2.33)	0.09	–	–
Ever had vaginal or anal sex with female partner	1.78 (1.12 to 2.82)	0.01	1.77 (1.10 to 2.83)	0.02
Community engagement in sexual health				
None	1	–	–	–
Minimal	0.48 (0.15 to 1.57)	0.22	–	–
Moderate	1.48 (0.73 to 3.00)	0.28	–	–
Substantial	1.68 (0.80 to 3.52)	0.17	–	–
HIV testing history				
Never	1	–	1	–
Self tested only	2.39 (1.38 to 4.14)	<0.01	1.62 (0.85 to 3.10)	0.15
Facility tested only	1.07 (0.45 to 2.52)	0.88	0.46 (0.13 to 1.59)	0.22
Both self and facility test	2.36 (1.38 to 4.03)	<0.01	1.57 (0.84 to 2.94)	0.16
High school education or lower (i.e. low education)	0.84 (0.53 to 1.31)	0.44	0.31 (0.11 to 0.83)	0.02
Interactions				
Never Tested × Higher education	–	–	1	–
Self tested × Low education	–	–	3.64 (1.00 to 13.25)	0.05
Facility tested × Low education	–	–	9.75 (1.53 to 62.02)	0.02
Both self‐ and facility tested × Low education	–	–	3.70 (1.05 to 13.10)	0.04
			Hosmer‐Lemeshow test χ^2^(6) = 1.18, *p* = 0.98	

AOR, adjusted odds ratio adjusted for ever had sex with female partner, and HIV testing history; 95% CI , 95% confidence interval.

**Table 4:** Odds of experiencing HIV test pressure according to HIV testing history, by education level of MSM in China (N* *=* *1044), 2017

	High school education or below		College education or above	
	AOR (95% CI)	*p* value	AOR (95% CI)	*p* value
HIV testing history				
Never tested	1	–	1	–
Self tested only	5.88 (1.92 to 17.99)	<0.01	1.62 (0.85 to 3.10)	0.15
Facility tested only	4.41 (1.11 to 17.59)	0.04	0.46 (0.13 to 1.58)	0.22
Both self and facility test	5.88 (1.96 to 17.64)	<0.01	1.58 (0.84 to 2.96)	0.15

AOR adjusted for ever had sex with female partner, and HIV testing history; 95% CI. AOR, adjusted odds ratio; 95% CI , 95% confidence interval.

### 3.3 Qualitative study results

Out of 96 men who reported experiencing pressured testing, 55 gave online consent for an individual follow‐up interview. We contacted all 55 eligible participants by phone, after which 17 agreed to be interviewed. None of the 12 participants who reported experiencing physical violence or felt they were being excessively controlled (12/55) agreed to be interviewed. The mean age of the participants was 27.5 (SD = 8.0), slightly older than the full cohort for pressure. Seven out of 17 (41.2%) men had college education or higher compared with 65% of the whole sample. Among all participants, 11 had a facility‐based test, 5 took a self‐test and 1 refused testing. The participant who eventually refused the test nevertheless reported feeling pressured because of the numerous attempts to make him take a test “in the name of love,” but the participant was not interested in an intimate relationship with the one who was pressuring him.

We found that participants had different understandings of pressure. First, “I did not actively want to [take the test] – I think this counts as some kind of pressured test.” (Qingdao, 40‐year old, postgraduate). Second, “my understanding is that when they want to develop a relationship, then they require each other to take a test. This is pressured testing.” (Guangzhou, 41‐year old, high school or below). Third, “pressure means a feeling of being forced by others, who are very determined, to take a test.” (Jining, 24 years old, university). Participants reported being initially unwilling to take a test due to either self‐perceived stigma associated with HIV testing or misperception of HIV risk. Consistent with the quantitative survey, the interviews revealed that the most common form of pressured testing was the threat of ending the relationship. Verbal abuse, psychological pressure and the threat of violence were also reported occasionally. Three participants were not informed about the test until they arrived at the testing facility. Boyfriends and sexual partners were the most frequent source of pressure, followed by close friends with no sexual relationship. Other people who pressured were healthcare workers and, in one case, a senior colleague. We examined pressured testing in terms of the pre‐test contexts, during‐test contexts, post‐test impact and overall attitudes to pressured testing.

### 3.4 Pre‐test contexts

Pressure occurred most frequently in the context of an existing, often intimate relationship with the one who pressured them. Some pressured cases happened at the point of or before unprotected sex. Others happened after unprotected anal sex with the participants (Qingdao, 40 years old, postgraduate; Qingdao, 20‐year old, high school or below). The participants were primarily pressured into taking a test by their sexual partners for reassurance that they were not at risk for acquiring HIV. Other cases of pressure involving sex often occurred at the beginning of a long‐term romantic relationship, and an HIV test was considered a “healthy start [for] a happy relationship” (Jinan, 26 years old, university).

Not being informed of the purpose of the upcoming test was a contextual contributor to the feeling of being pressured. Most men had initial negative feelings towards pressured HIV testing such as feeling angry, distrusted, discriminated against and humiliated. Poor explanations about HIV and the necessity of HIV testing contributed to negative feelings. A few participants described that they were only informed about the purpose of the test after it was done. In these cases, men reported that being aware of the purpose of the test would reduce their perception of feeling pressured: “I don't even know what advantages the test may bring me. I certainly cannot accept it. If you provide a bit more relevant knowledge, it might be easier for me to accept it and [it] would not feel too pressured.” (Jiangmen, 20 years old, some college). One man was threatened by a doctor that he would be refused treatment if he did not get tested: “if you have HIV and I do the surgery on you, I might get infected if I cut myself” (Shenzhen, 46‐year old, high school or below). And the power imbalance between the participant and the doctor put him in a vulnerable position: “How could we patients argue with a doctor? I only wished he would treat me well and I must be nice to him.” (Shenzhen, 46‐year old, high school or below).

### 3.5 During‐test contexts

Among the 16 participants who took a test after being pressured, the tension between the participant and the one who pressured eased during the test process (from entering the facility or test initiation to result release). Communicating with doctors was a major factor that mitigated the feeling of pressure and improved participants’ willingness to test: “Since I saw the doctor, my attitudes [towards HIV testing] changed because he did not view me differently and he treated us patients equally.” (Jining, 22 years old, some college). All of our participants tested HIV‐negative and this assured both the participant and the one who pressured them. One participant said “I was very happy when I got my result. Life is full of sunshine!” (Qingdao, 40 years old, postgraduate). Another mitigating factor was the intention of the test as perceived by the participant: “When he dragged me into the hall of [the CDC], I felt really embarrassed. I felt pressured. But then I felt happy at the same time because there is someone who really cares about me and always reminds me. I think it's a sweet pressured test” (Jinan, 26‐year old, university).

### 3.6 Post‐test impact

We observed four major changes as a result of the pressured HIV test. These changes included changes in attitude towards HIV testing, changes in testing behaviours, changes in sexual behaviours and changes in the relationship between the participant and the one who pressured him. For several participants, the pressured test was their first HIV test and they became more receptive to HIV testing afterwards. We identified two reasons for this attitude change. First, undergoing one HIV test increased the participant's HIV awareness and the need to test for HIV. One participant remarked “I didn't know about [HIV risk] but he made me aware of it” and “after the test, I started to search for relevant information” (Jiangmen, 20 years old, some college). Second, a positive experience going through the process of testing reduced test‐associated fears.

Among participants who had never received an HIV test before their pressure experience, testing behaviours also improved. After the pressured test, some participants developed a pattern of regular testing. Among those who received the pressured test at a facility, a few have done regular self‐tests since then. “Afterwards, I started to take regular tests. I take self‐tests, and do not go to hospitals to take a test.” (Jiangmen, 20 years old, some college). Moreover, participants reported more frequent condom use during sex following their experience of the pressured test. This was due to increased awareness of HIV risk. For participants who were already well aware of HIV infection risk among MSM, attitudes and testing behaviours remained largely the same.

We also recognized changes in the dynamics of relationships as a result of the pressured testing experience. Many participants admitted that their relationship became closer, with more mutual trust, emotional support and communication. They commented that “we have more harmonious relationship because he trusted me more” (Shenzhen, 30 years old, high school or below) and “[our relationship] became better because life was full of sunshine. We do not have anything to worry about now” (Qingdao, 40‐year old, postgraduate). One regarded the pressured experience as an expression of love and felt happy afterwards (Jinan, 26‐year old, university). Those who reported a better relationship appear to have experienced pressure mainly due to miscommunications such as poor explanations and not being informed about the test. However, a couple of participants broke up with their partners after being pressured because they witnessed an aggressive side of their partners, “I changed my views towards him. I did not know that he had a side like that. Then I started to keep my distance from him.”(Qingdao, 20‐year old, high school or below). In cases where the participant witnessed an aggressive side of the one who pressured them, their mutual relationship worsened following the pressured event.

### 3.7 Attitudes towards pressured test

Tolerance of pressured testing also depended on the participant's relationship with the one who pressured them. To some participants, pressure by family members and intimate partners was acceptable, but pressure by other people was not. Chinese cultural customs such as filial piety (i.e. respect for one's parents and elders) may play a role in participants’ perceptions of their sexual orientation and lead to a higher tolerance of pressure by family members. For example, one participant remarked “I can accept if my family coerces me to take [the test], even if they physically or verbally abuse me. I feel sorry for my parents and guilty because I am gay and cannot continue the family line” and “I can also accept if my boyfriend does this because he has good intentions for my health after all. So this is acceptable too” (Jiangmen, 33 years old, university). However, the same participant perceived pressured HIV testing by other friends as a violation of human rights that he had no obligation to submit to: “If it is my friend, I think, firstly, it's unnecessary. Second, he doesn't have the right to force me do anything. I am not bound to his intentions” (Jiangmen, 33 years old, university). A test ordered by healthcare providers before initiating treatment, even if pressured, was perceived as “possibly a necessary test”. (Shenzhen, 46‐year old, high school or below).

In addition, when asked whether they would ever coerce others to take the test, some participants explicitly expressed that “It depends on my relationship with him. If we are really good friends, I would pressure him to take the test. If he is still unwilling to, it is unnecessary to keep such a friend because friends like this are dangerous” (Jiangmen, 33‐year old, university). On further clarification, ‘really good friends’ can refer both a non‐sexual or sexual relationship. Others remarked they would not force others to take a test. “I don't think pressure is a good way because making people do something is not pleasant. We wouldn't feel happy. I may try to persuade someone but whether they do it or not is their own business. I won't force them to do anything they don't want to do” (Shenzhen, 30‐year old, high school or below).

## 4 Discussion

The WHO declares that HIV tests should be voluntary and ‘mandatory or compulsory (coerced) testing is never appropriate, regardless of where that coercion comes from: healthcare providers, partners, family members, employers, or others’^2^. We found that the experience of HIV test pressure was relatively common among Chinese MSM, but it was not necessarily perceived by Chinese MSM as a harmful or unacceptable act. Perceptions of what constitutes pressure existed on a continuum and the acceptance of pressure depended on the participant's relationship with the one who pressured him to test. We observed increased rates of pressure among men of low education levels who only used HIV self‐testing, but not among men of high education levels who used HIV self‐testing. Monitoring for the potential harms of HIV self‐testing is particularly important as HIV self‐testing becomes increasingly scaled up and decentralized, and an in‐depth understanding of the social and educational contexts underlying pressure may help reduce pressured testing while enhancing HIV test uptake.

We found that HIV test pressure was common among Chinese MSM. This is consistent with the Chinese 7, 19 and global literature 10 on HIV test coercion. The quantitative data revealed that most perpatrators were most commonly regular or casual sex partners, followed by friends. The interviews provided insights into this finding. Based on our participants’ perceptions, the person who pressured them appeared to be aware of a higher risk of HIV infection among MSM. Persuading the participant to get tested was mainly for the protection of the person who pressured before they entered into a sexual or romantic relationship with the participant. Although not explicitly asked, this may be within the context of negotiated safety that is, agreement between sexual partners to use condoms during sex until ‘safety’ from HIV is established, which includes the awareness of HIV serostatus and negotiation of types of sexual practices within and outside their relationship 9. Friends initiating pressure was perceived by participants to be a caring act out of concern for their health. Participants found pressure more acceptable when it was initiated by someone close to them. A common problem that emerged was miscommunication between participants and the one who pressured them. Lack of advanced notice of the test, poor explanations about HIV and the HIV test, and inadequate information provision frequently emerged as a cause of unpleasant feelings such as anger and humiliation in participants who felt they were being pressured. Therefore, improving communication between relevant parties may help to prevent test pressure, particularly in scenarios of decentralized testing. Furthermore, in contexts of negotiated safety, it is important to equip men to effectively and non‐coercively communicate the importance of testing to establish the serostatus within sexual partnerships 9.

Our study found increased likelihood of pressure amongst men with lower education who only used HIV self‐testing. It is possible that men with higher educational attainment are better able to advocate for themselves in the dynamics of their relationship 20, and may therefore be more resistant to pressure. In addition, the decentralized nature of HIVST may facilitate pressure amongst vulnerable populations (e.g. those with high school education or lower) compared to facility‐based tests if a health‐professional is not involved in the testing process. To date, this finding has not been reported in the limited post‐HIVST surveillance literature 21. However, most studies provide supervised self‐testing and the group that asks about pressure is also the same group that provides the testing. Furthermore research regarding the link between HIVST and pressured testing is needed. The in‐depth interviews revealed that there were unmeasured factors in the quantitative survey that were important in influencing the likelihood of pressure aside from the type of test, such as the miscommunication of intentions and information regarding HIV and its testing process, attitudes (altruistic to selfish) and power imbalance (vulnerable to dominant) from those who were pressuring these men to test.

Accurately measuring pressure is challenging as the experience and acceptability of pressure are context‐dependent. Both quantitative and qualitative data revealed that after pressured testing, the majority of participants tested again for HIV without further pressure, and those who had not yet tested again intended to test. Furthermore, most participants experienced positive changes of attitudes towards HIV testing and sexual behaviours. Men who were pressured became more attuned to the importance of HIV testing and safe sex in preventing HIV infection. This may be due to increased knowledge of HIV, more awareness of the need to test for HIV, and reduced self‐perceived social stigma as a result of their testing experience in HIV testing facilities. We also found that relationships actually improved for participants who perceived that the one who pressured them had good intentions. However, these positive changes do not justify pressure. Balancing autonomy in HIV testing with the relational responsibility of HIV sero‐status disclosure should be a consideration.

To our knowledge, this is the first study to explore the frequency of pressured HIV testing and its contexts as well as consequences among Chinese MSM. We adopted a mixed methods approach using quantitative study questions to determine the extent of and associations with HIV test pressure experience, and a follow‐up qualitative study to further understand perceptions of pressure and its mechanisms, processes, and consequences. However, our study also had several limitations First, we did not interview individuals who pressured other people to test. It is important to explore reasons behind pressured testing, in particular, the motivations behind pressuring others to take HIV test in future research. For instance, we did not explicitly ask men if pressured testing was in the context of negotiated safety. It would be important for future studies to distinguish inappropriate pressure to test from pressure to test motivated by self‐protection within a sexual relationship. Apart from assessing underlying motivations to pressure, the appropriateness of various forms of pressure should also be explored as the boundaries between appropriate and inappropriate pressure to test can be blurry. Second, because the survey was a self‐administered questionnaire, social desirability bias may exist. However, our study was a computer‐based online survey, and we anticipated that this bias would be minor. Third, there may be a possibility for response bias regarding the man's experience of pressured testing being influenced by the outcome of the HIV test. All men who participated in the in‐depth interviews self‐reported as HIV‐negative.

Nearly one in ten Chinese MSM reported experiencing unwanted pressure to test for HIV. However, there was a wide spectrum in the types of pressure experienced, initiated by a variety of individuals, though most had close relationships with the participants. While the experience of pressure was perceived to be negative, it resulted in a positive change in testing behaviours for the majority of participants interviewed. Careful consideration should be given to further understand the social contexts of pressure, which may influence how men perceive and react to pressure.

## Competing interests

All authors declare they do not have any competing interests.

## Authors’ contributions

JDT, JJO, WD and ND contributed to the conception and design of the study. DW, WH, CW and BY provided oversight for data collection. DW, WH, WM, DK, ML, GM, LY and SH assisted in the data collection. JJO, WD analysed the data and drafted the paper. All authors revised the manuscript and approved the final version to be published.
